# *N*,*N*′-Dibenzyl-2,2′-(3,6-dioxaoctane-1,8-diyldi­oxy)dibenzamide

**DOI:** 10.1107/S1600536811002212

**Published:** 2011-01-22

**Authors:** Yong-Hong Wen, Ji-Min Dai

**Affiliations:** a College of Chemistry and Molecular Engineering, Qingdao University of Science and Technology, Qingdao 266042, People’s Republic of China

## Abstract

The title compound, C_34_H_36_N_2_O_6_, located on a center of inversion, crystallizes with one half-mol­ecule in the asymmetric unit. The dihedral angle between the benzene rings is 86.19 (2)°. An intra­molecular N—H⋯O hydrogen bond forms a six-membered ring; it affects the conformation of the mol­ecule which adopts a folded rather than open conformation. The crystal packing is stabilized by inter­molecular C—H⋯O inter­actions.

## Related literature

For background to the applications of amide-type acyclic polyethers, see: Wen *et al.* (2002 ▶, 2008 ▶); Lehn *et al.* (1995 ▶). For related structures of amide-type acyclic polyethers, see: Wen *et al.* (2005 ▶, 2008 ▶).
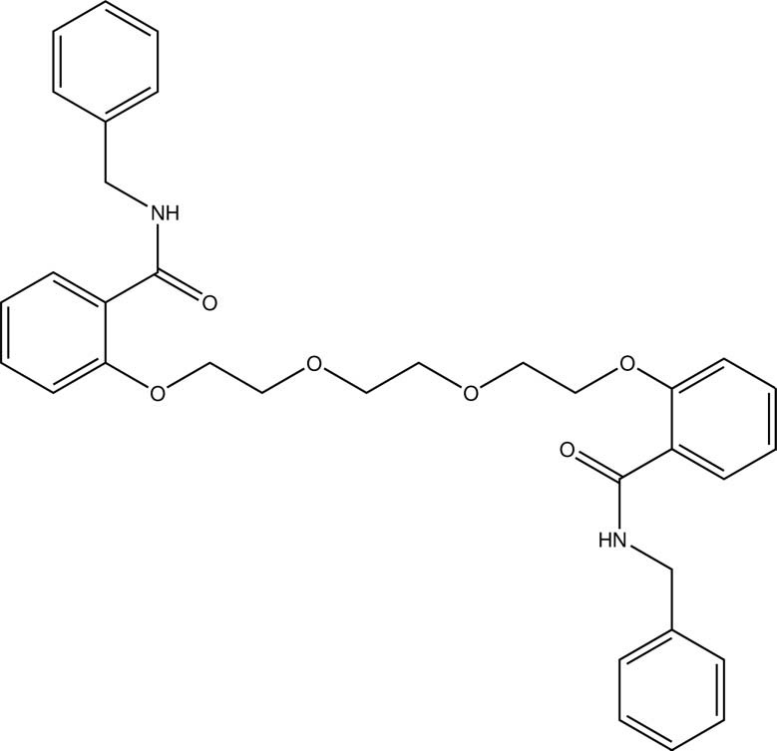


## Experimental

### 

#### Crystal data

C_34_H_36_N_2_O_6_
*M* *_r_* = 568.65
Monoclinic, 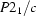

*a* = 12.065 (3) Å
*b* = 15.964 (4) Å
*c* = 8.251 (2) Å
β = 104.820 (5)°
*V* = 1536.3 (7) Å^3^
*Z* = 2
Mo *K*α radiation
μ = 0.08 mm^−1^
*T* = 294 K
0.24 × 0.20 × 0.16 mm

#### Data collection

Bruker SMART CCD area-detector diffractometer
Absorption correction: multi-scan (*SADABS*; Sheldrick, 1996 ▶) *T*_min_ = 0.976, *T*_max_ = 0.983
7850 measured reflections
2707 independent reflections
1284 reflections with *I* > 2σ(*I*)
*R*_int_ = 0.041

#### Refinement

*R*[*F* ^2^ > 2σ(*F* ^2^)] = 0.048
*wR*(*F* ^2^) = 0.149
*S* = 1.00
2707 reflections
190 parameters
36 restraints
H-atom parameters constrained
Δρ_max_ = 0.21 e Å^−3^
Δρ_min_ = −0.17 e Å^−3^

### 

Data collection: *SMART* (Bruker, 2001 ▶); cell refinement: *SAINT* (Bruker, 2001 ▶); data reduction: *SAINT*; program(s) used to solve structure: *SHELXTL* (Sheldrick, 2008 ▶); program(s) used to refine structure: *SHELXTL*; molecular graphics: *SHELXTL*; software used to prepare material for publication: *SHELXTL*.

## Supplementary Material

Crystal structure: contains datablocks I, global. DOI: 10.1107/S1600536811002212/bv2169sup1.cif

Structure factors: contains datablocks I. DOI: 10.1107/S1600536811002212/bv2169Isup2.hkl

Additional supplementary materials:  crystallographic information; 3D view; checkCIF report

## Figures and Tables

**Table 1:** Hydrogen-bond geometry (Å, °)

*D*—H⋯*A*	*D*—H	H⋯*A*	*D*⋯*A*	*D*—H⋯*A*
N1—H1⋯O2	0.86	1.97	2.645 (3)	135
C15—H15*B*⋯O1^i^	0.97	2.69	3.580 (5)	152

Symmetry code: (i) 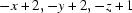.

**Fig. 1.**
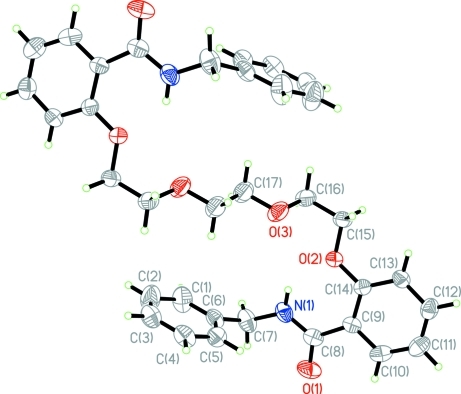
The molecular structure of the title compound, with atom labels and 30% probability displacement ellipsoids, Unlabelled atoms are related to labelled atoms by the symmetry operator (1 - x,2 - y,-z).

**Fig. 2.**
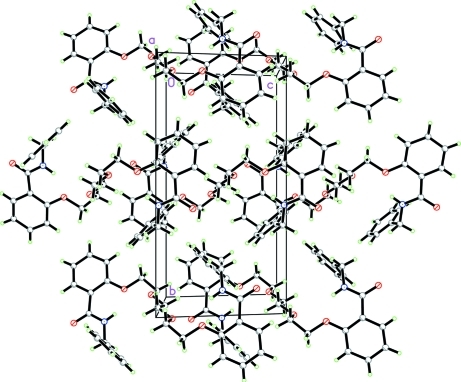
The packing diagram of the title compound, viewed down the a axis.

## supplementary crystallographic information

### Comment

Increasing attention has focused on acyclic polyether compounds, due to their complexing ability (Wen *et al.*, 2008), selectivity (Wen *et al.*, 2002) to metal ions and their potential application in supramolecular chemistry (Lehn *et al.*, 1995). In our ongoing studies of structures and properties of diamide-type acyclic polyethers, a new flexible acyclic polyether ligand 3,6-dioxa-1,8-octylenedi(*N*-benzyl-salicylamide) was synthesized. Herein we report the synthesis and structure of the title compound, (Fig. 1).

The title compound crystallizes in the monoclinic space group *P*2_1_/*c*. The asymmetric unit of the title compound contains one half-molecule, the other half being related by a crystallographic center of inversion (Fig. 1). All bond lengths and angles in the title compound are within normal ranges, and comparable with those in the related compounds (Wen *et al.*, 2005, & Wen *et al.*, 2008). In the asymmetric unit, the dihedral angle between two benzene rings is 86.19 (2)°. An intramolecular N(1)—H(1)···O(2) hydrogen bond (Table 1) forms a six-numbered ring, and affects the conformation of the molecule which thus adopts a folded rather than open conformation. The crystal packing is stabilized by intermolecular C(15)—H(15B)···O(1) short-contact interactions (Table 1).

### Experimental

The title compound was synthesized according to literature method (Wen *et al.*, 2008). Colourless single cystals suitable for X-ray diffraction were obtained by slow evaporation of an ethanol solution over a period of 5 d.

### Refinement

H atoms were positioned geometrically, with N—H = 0.86 Å and C—H = 0.95–0.99 Å, respectively, and constrained to ride on their parent atoms, with *U*_iso_(H) = 1.2 *U*_eq_(C,*N*). In the refinement, SIMU was used to restraint the displacement parameters of the atoms N1, C7, C6, C5, C1, C2 and C3 to move similarly.

### Crystal data

**Table:**

C_34_H_36_N_2_O_6_	*F*(000) = 604
*M**_r_* = 568.65	*D*_x_ = 1.229 Mg m^−^^3^
Monoclinic, *P*2_1_/*c*	Mo *K*α radiation, λ = 0.71073 Å
Hall symbol: -P 2ybc	Cell parameters from 1307 reflections
*a* = 12.065 (3) Å	θ = 2.6–20.3°
*b* = 15.964 (4) Å	µ = 0.08 mm^−^^1^
*c* = 8.251 (2) Å	*T* = 294 K
β = 104.820 (5)°	Prism, colourless
*V* = 1536.3 (7) Å^3^	0.24 × 0.20 × 0.16 mm
*Z* = 2	

### Data collection

**Table:**

Bruker SMART CCD area-detector diffractometer	2707 independent reflections
Radiation source: fine-focus sealed tube	1284 reflections with *I* > 2σ(*I*)
graphite	*R*_int_ = 0.041
φ and ω scans	θ_max_ = 25.0°, θ_min_ = 1.8°
Absorption correction: multi-scan (*SADABS*; Sheldrick, 1996)	*h* = −14→14
*T*_min_ = 0.976, *T*_max_ = 0.983	*k* = −18→18
7850 measured reflections	*l* = −7→9

### Refinement

**Table:**

Refinement on *F*^2^	Primary atom site location: structure-invariant direct methods
Least-squares matrix: full	Secondary atom site location: difference Fourier map
*R*[*F*^2^ > 2σ(*F*^2^)] = 0.048	Hydrogen site location: inferred from neighbouring sites
*wR*(*F*^2^) = 0.149	H-atom parameters constrained
*S* = 1.00	*w* = 1/[σ^2^(*F*_o_^2^) + (0.0574*P*)^2^ + 0.3181*P*] where *P* = (*F*_o_^2^ + 2*F*_c_^2^)/3
2707 reflections	(Δ/σ)_max_ < 0.001
190 parameters	Δρ_max_ = 0.21 e Å^−^^3^
36 restraints	Δρ_min_ = −0.17 e Å^−^^3^

### Special details

**Table:**

Geometry. All e.s.d.'s (except the e.s.d. in the dihedral angle between two l.s. planes) are estimated using the full covariance matrix. The cell e.s.d.'s are taken into account individually in the estimation of e.s.d.'s in distances, angles and torsion angles; correlations between e.s.d.'s in cell parameters are only used when they are defined by crystal symmetry. An approximate (isotropic) treatment of cell e.s.d.'s is used for estimating e.s.d.'s involving l.s. planes.
Refinement. Refinement of *F*^2^ against ALL reflections. The weighted *R*-factor *wR* and goodness of fit *S* are based on *F*^2^, conventional *R*-factors *R* are based on *F*, with *F* set to zero for negative *F*^2^. The threshold expression of *F*^2^ > σ(*F*^2^) is used only for calculating *R*-factors(gt) *etc*. and is not relevant to the choice of reflections for refinement. *R*-factors based on *F*^2^ are statistically about twice as large as those based on *F*, and *R*- factors based on ALL data will be even larger.

### Fractional atomic coordinates and isotropic or equivalent isotropic displacement parameters (Å^2^)

**Table:**

	*x*	*y*	*z*	*U*_iso_*/*U*_eq_	
O1	0.8814 (2)	0.90823 (13)	0.7723 (3)	0.1004 (8)	
O2	0.83284 (14)	1.05219 (11)	0.3438 (2)	0.0662 (5)	
O3	0.63670 (17)	1.03915 (16)	0.0958 (3)	0.0971 (8)	
N1	0.7835 (2)	0.91898 (14)	0.5028 (3)	0.0738 (7)	
H1	0.7665	0.9504	0.4152	0.089*	
C1	0.5392 (3)	0.7869 (2)	0.3307 (5)	0.1097 (13)	
H1A	0.5756	0.7521	0.2699	0.132*	
C2	0.4202 (4)	0.7860 (3)	0.2970 (5)	0.1218 (14)	
H2	0.3780	0.7504	0.2147	0.146*	
C3	0.3657 (3)	0.8361 (2)	0.3827 (5)	0.0925 (10)	
H3	0.2861	0.8355	0.3603	0.111*	
C4	0.4281 (3)	0.88690 (19)	0.5011 (4)	0.0820 (10)	
H4	0.3913	0.9219	0.5611	0.098*	
C5	0.5463 (3)	0.88771 (18)	0.5347 (4)	0.0750 (9)	
H5	0.5878	0.9235	0.6172	0.090*	
C6	0.6031 (3)	0.83769 (17)	0.4504 (4)	0.0670 (8)	
C7	0.7328 (3)	0.83619 (18)	0.4915 (5)	0.0901 (10)	
H7B	0.7619	0.8074	0.5974	0.108*	
H7A	0.7564	0.8045	0.4057	0.108*	
C8	0.8553 (3)	0.94947 (19)	0.6421 (4)	0.0679 (8)	
C9	0.9030 (2)	1.03553 (16)	0.6358 (3)	0.0562 (7)	
C10	0.9664 (3)	1.0684 (2)	0.7883 (4)	0.0741 (9)	
H10	0.9754	1.0363	0.8848	0.089*	
C11	1.0157 (3)	1.1460 (2)	0.8009 (4)	0.0831 (10)	
H11	1.0576	1.1658	0.9045	0.100*	
C12	1.0031 (2)	1.1942 (2)	0.6606 (4)	0.0752 (9)	
H12	1.0360	1.2472	0.6687	0.090*	
C13	0.9419 (2)	1.16458 (18)	0.5074 (4)	0.0644 (8)	
H13	0.9334	1.1978	0.4124	0.077*	
C14	0.8926 (2)	1.08525 (17)	0.4938 (3)	0.0549 (7)	
C15	0.8169 (2)	1.09965 (18)	0.1928 (3)	0.0706 (8)	
H15B	0.8901	1.1111	0.1691	0.085*	
H15A	0.7796	1.1525	0.2029	0.085*	
C16	0.7438 (2)	1.0481 (2)	0.0572 (4)	0.0816 (9)	
H16B	0.7341	1.0755	−0.0504	0.098*	
H16A	0.7786	0.9936	0.0524	0.098*	
C17	0.5563 (2)	0.9992 (3)	−0.0245 (4)	0.1032 (12)	
H17A	0.5797	0.9418	−0.0352	0.124*	
H17B	0.5477	1.0270	−0.1316	0.124*	

### Atomic displacement parameters (Å^2^)

**Table:**

	*U*^11^	*U*^22^	*U*^33^	*U*^12^	*U*^13^	*U*^23^
O1	0.1298 (19)	0.0877 (16)	0.0854 (16)	0.0126 (13)	0.0307 (15)	0.0363 (13)
O2	0.0697 (12)	0.0723 (12)	0.0529 (12)	−0.0104 (10)	0.0088 (10)	0.0102 (10)
O3	0.0592 (12)	0.166 (2)	0.0638 (13)	−0.0177 (13)	0.0111 (11)	−0.0132 (14)
N1	0.0729 (15)	0.0547 (15)	0.094 (2)	−0.0021 (12)	0.0223 (15)	0.0136 (13)
C1	0.104 (3)	0.111 (3)	0.135 (3)	−0.023 (2)	0.070 (3)	−0.057 (3)
C2	0.109 (3)	0.143 (4)	0.123 (3)	−0.039 (3)	0.048 (3)	−0.060 (3)
C3	0.078 (2)	0.095 (3)	0.112 (3)	−0.005 (2)	0.038 (2)	0.004 (2)
C4	0.093 (3)	0.063 (2)	0.104 (3)	0.0074 (18)	0.052 (2)	0.0007 (19)
C5	0.088 (2)	0.060 (2)	0.082 (2)	0.0002 (16)	0.0303 (19)	−0.0095 (15)
C6	0.080 (2)	0.0470 (17)	0.084 (2)	−0.0002 (15)	0.0404 (18)	0.0021 (15)
C7	0.082 (2)	0.061 (2)	0.136 (3)	0.0042 (17)	0.042 (2)	0.0050 (19)
C8	0.0675 (18)	0.069 (2)	0.072 (2)	0.0162 (16)	0.0270 (17)	0.0122 (18)
C9	0.0540 (15)	0.0587 (17)	0.0592 (18)	0.0141 (13)	0.0206 (14)	0.0100 (14)
C10	0.085 (2)	0.082 (2)	0.056 (2)	0.0255 (18)	0.0178 (17)	0.0048 (17)
C11	0.085 (2)	0.090 (3)	0.072 (2)	0.015 (2)	0.0155 (19)	−0.021 (2)
C12	0.0698 (19)	0.075 (2)	0.083 (2)	−0.0045 (16)	0.0235 (18)	−0.021 (2)
C13	0.0664 (17)	0.0639 (19)	0.068 (2)	−0.0026 (15)	0.0261 (16)	0.0025 (15)
C14	0.0453 (14)	0.0676 (19)	0.0531 (17)	0.0065 (13)	0.0152 (13)	0.0018 (14)
C15	0.0653 (18)	0.087 (2)	0.0589 (18)	−0.0016 (16)	0.0151 (15)	0.0157 (16)
C16	0.0552 (18)	0.129 (3)	0.060 (2)	−0.0024 (18)	0.0141 (16)	0.0139 (19)
C17	0.077 (2)	0.161 (3)	0.071 (2)	−0.031 (2)	0.0201 (19)	−0.019 (2)

### Geometric parameters (Å, °)

**Table:**

O1—C8	1.231 (3)	C7—H7A	0.9700
O2—C14	1.369 (3)	C8—C9	1.496 (4)
O2—C15	1.429 (3)	C9—C14	1.394 (3)
O3—C17	1.356 (3)	C9—C10	1.397 (4)
O3—C16	1.415 (3)	C10—C11	1.365 (4)
N1—C8	1.341 (3)	C10—H10	0.9300
N1—C7	1.449 (3)	C11—C12	1.366 (4)
N1—H1	0.8600	C11—H11	0.9300
C1—C6	1.356 (4)	C12—C13	1.374 (4)
C1—C2	1.392 (5)	C12—H12	0.9300
C1—H1A	0.9300	C13—C14	1.391 (4)
C2—C3	1.345 (4)	C13—H13	0.9300
C2—H2	0.9300	C15—C16	1.484 (4)
C3—C4	1.343 (4)	C15—H15B	0.9700
C3—H3	0.9300	C15—H15A	0.9700
C4—C5	1.381 (4)	C16—H16B	0.9700
C4—H4	0.9300	C16—H16A	0.9700
C5—C6	1.355 (4)	C17—C17^i^	1.514 (6)
C5—H5	0.9300	C17—H17A	0.9700
C6—C7	1.514 (4)	C17—H17B	0.9700
C7—H7B	0.9700		
			
C14—O2—C15	120.4 (2)	C10—C9—C8	116.2 (3)
C17—O3—C16	113.9 (2)	C11—C10—C9	122.5 (3)
C8—N1—C7	123.7 (3)	C11—C10—H10	118.8
C8—N1—H1	118.1	C9—C10—H10	118.8
C7—N1—H1	118.1	C10—C11—C12	119.6 (3)
C6—C1—C2	121.1 (3)	C10—C11—H11	120.2
C6—C1—H1A	119.4	C12—C11—H11	120.2
C2—C1—H1A	119.4	C11—C12—C13	120.2 (3)
C3—C2—C1	120.5 (3)	C11—C12—H12	119.9
C3—C2—H2	119.8	C13—C12—H12	119.9
C1—C2—H2	119.8	C12—C13—C14	120.3 (3)
C4—C3—C2	118.9 (3)	C12—C13—H13	119.8
C4—C3—H3	120.5	C14—C13—H13	119.8
C2—C3—H3	120.5	O2—C14—C13	122.6 (2)
C3—C4—C5	120.6 (3)	O2—C14—C9	117.0 (2)
C3—C4—H4	119.7	C13—C14—C9	120.4 (3)
C5—C4—H4	119.7	O2—C15—C16	106.5 (2)
C6—C5—C4	121.6 (3)	O2—C15—H15B	110.4
C6—C5—H5	119.2	C16—C15—H15B	110.4
C4—C5—H5	119.2	O2—C15—H15A	110.4
C5—C6—C1	117.3 (3)	C16—C15—H15A	110.4
C5—C6—C7	121.6 (3)	H15B—C15—H15A	108.6
C1—C6—C7	121.1 (3)	O3—C16—C15	106.7 (2)
N1—C7—C6	113.2 (2)	O3—C16—H16B	110.4
N1—C7—H7B	108.9	C15—C16—H16B	110.4
C6—C7—H7B	108.9	O3—C16—H16A	110.4
N1—C7—H7A	108.9	C15—C16—H16A	110.4
C6—C7—H7A	108.9	H16B—C16—H16A	108.6
H7B—C7—H7A	107.7	O3—C17—C17^i^	108.6 (3)
O1—C8—N1	121.3 (3)	O3—C17—H17A	110.0
O1—C8—C9	120.5 (3)	C17^i^—C17—H17A	110.0
N1—C8—C9	118.3 (3)	O3—C17—H17B	110.0
C14—C9—C10	117.0 (3)	C17^i^—C17—H17B	110.0
C14—C9—C8	126.8 (3)	H17A—C17—H17B	108.3
			
C6—C1—C2—C3	0.3 (6)	C14—C9—C10—C11	0.7 (4)
C1—C2—C3—C4	0.0 (6)	C8—C9—C10—C11	179.5 (3)
C2—C3—C4—C5	−0.1 (5)	C9—C10—C11—C12	0.2 (5)
C3—C4—C5—C6	−0.1 (5)	C10—C11—C12—C13	−0.5 (5)
C4—C5—C6—C1	0.4 (5)	C11—C12—C13—C14	−0.2 (4)
C4—C5—C6—C7	−177.7 (3)	C15—O2—C14—C13	−1.1 (4)
C2—C1—C6—C5	−0.5 (5)	C15—O2—C14—C9	179.1 (2)
C2—C1—C6—C7	177.6 (3)	C12—C13—C14—O2	−178.7 (2)
C8—N1—C7—C6	119.0 (3)	C12—C13—C14—C9	1.2 (4)
C5—C6—C7—N1	−48.7 (4)	C10—C9—C14—O2	178.5 (2)
C1—C6—C7—N1	133.4 (3)	C8—C9—C14—O2	−0.2 (4)
C7—N1—C8—O1	−1.2 (4)	C10—C9—C14—C13	−1.3 (4)
C7—N1—C8—C9	179.0 (2)	C8—C9—C14—C13	180.0 (2)
O1—C8—C9—C14	171.6 (3)	C14—O2—C15—C16	−176.8 (2)
N1—C8—C9—C14	−8.5 (4)	C17—O3—C16—C15	174.7 (3)
O1—C8—C9—C10	−7.1 (4)	O2—C15—C16—O3	63.9 (3)
N1—C8—C9—C10	172.7 (2)	C16—O3—C17—C17^i^	−174.9 (4)

Symmetry codes: (i) −*x*+1, −*y*+2, −*z*.

### Hydrogen-bond geometry (Å, °)

**Table:**

*D*—H···*A*	*D*—H	H···*A*	*D*···*A*	*D*—H···*A*
N1—H1···O2	0.86	1.97	2.645 (3)	135
C15—H15B···O1^ii^	0.97	2.69	3.580 (5)	152

Symmetry codes: (ii) −*x*+2, −*y*+2, −*z*+1.
